# The Anti-Staphylococcal Potential of Ethanolic Polish Propolis Extracts

**DOI:** 10.3390/molecules24091732

**Published:** 2019-05-03

**Authors:** Katarzyna Grecka, Piotr M. Kuś, Piotr Okińczyc, Randy W. Worobo, Justyna Walkusz, Piotr Szweda

**Affiliations:** 1 Department of Pharmaceutical Technology and Biochemistry, Faculty of Chemistry, Gdańsk University of Technology, ul. G. Narutowicza 11/12, 80-233 Gdańsk, Poland; kagrecka@gmail.com (K.G.); jusgry@wp.pl (J.W.); 2 Department of Pharmacognosy and Herbal Medicines, Wrocław Medical University, ul. Borowska 211a, 50-556 Wrocław, Poland; kus.piotrek@gmail.com (P.M.K.); piotrokinczyc@gmail.com (P.O.); 3 Department of Food Science, Cornell University, Ithaca, NY 14853, USA; rww8@cornell.edu

**Keywords:** propolis, *S. aureus*, biofilm, synergism, flavonoids

## Abstract

The principal objective of this study was to determine the anti-staphylococcal potential of ethanol extracts of propolis (EEPs). A total of 20 samples of propolis collected from apiaries located in different regions of Poland were used in the study. The two-fold broth microdilution method revealed some important differences in the antimicrobial activity of investigated EEPs. Up to the concentration of 4096 µg/mL no activity was observed against Gram-negative bacteria (*E. coli* and *P. aeruginosa*). Staphylococci exhibited much higher susceptibility. The highest efficiency observed for EEP12 and EEP20 (MIC values ranged between 32 and 256 µg/mL). However, the achievement of bactericidal effect usually required higher concentrations. In the case of clinical isolates of *S. aureus* MBC values for EEP12 and EEP20 ranged from 512 to 1024 µg/mL. The HPLC analysis revealed that these two products contained a higher concentration of flavonoids (flavonols, flavones, and flavanones) compared to other investigated EEPs. In checkerboard test, a synergistic anti-staphylococcal effect was observed for the action of EEP20 in combination with amikacin, kanamycin, gentamycin, tetracycline, and fusidic acid (all these antibiotics inhibit protein synthesis). Moreover, the investigated EEPs effectively eradicated staphylococcal biofilm. The obtained results clearly confirm the high anti-staphylococcal potential of propolis harvested in Polish apiaries.

## 1. Introduction

Based on the World Health Organization reports, the resistance of bacteria to well-known antibiotics is becoming a major global health challenge [1]. One of the groups of bacteria which have evolved mechanisms of resistance to a plethora of antibiotics currently in use for human and animal therapies are staphylococci, particularly *Staphylococcus aureus*. They are responsible for a broad spectrum of difficult to treat diseases including skin and ocular infections, foodborne illness, pneumonia, meningitis, endocarditis, and osteomyelitis. High pathogenicity of *S. aureus* is based on the production of a wide array of virulence factors that include protein A, coagulase, collagenase, hyaluronidase, hemolysins, lipases, different toxins, adhesive proteins and also proteins affecting the biofilm formation. These bacteria are very ubiquitous in the environment. Moreover, it colonizes approximately 30% of all humans—usually asymptomatically, however, any insufficiency of the host’s immune system poses a risk of infection development [2,3]. Due to the growing frequency of isolation of staphylococcal strains resistant to currently used antibiotics, their high virulence potential and common presence in environmental, there is an urgent need to search for new agents as well as therapy systems effective against these bacteria. The most promising alternative, non-antibiotic agents that exhibit anti-staphylococcal (including MRSA isolates) activity include: bacteriocins [4,5], bacteriophages [6,7], peptidoglycan hydrolases [8] compounds of plant origin - plant extracts, essential oils and their components [9,10,11,12] and silver nanoparticles [9,13]. Selection of resistant strains can be limited by combined therapy—simultaneous use of at least two antibiotics/agents that affect different molecular targets [14,15]. Another interesting approach for the treatment of infectious disease is photodynamic therapy [16,17] and some research groups developed new vaccines that seem to be effective in the prophylaxis of staphylococcal infections [18,19,20].

A promising but still an underestimated group of potential antimicrobial agents are bee products, especially bee propolis [11,21]. It is a highly agglutinative, resinous substance of complex chemical composition, that is collected by bees from flower and leaf buds. Some of its ingredients, mainly polyphenols, and flavonoids, exhibit high antimicrobial activity. As a consequence, bees use this product for the protection against dangerous pathogens from the hive environment [22]. Because of its’ antimicrobial potential propolis became one of the most common and important agents used in folk medicine in different regions of the world for the treatment of infections [23]. The chemical composition of raw propolis depends on many factors, among them the geographical location of apiary—species of plants that are available for bees, and environmental conditions (e.g., weather, the season of the year) are the most important. In general, it consists of 50% resin, 30% vegetable and bee wax, 10% essential oils, 5% pollen and 5% various other substances, including organic pollutants [24]. Propolis-containing extracts exhibit broad spectrum biological activity, including antiseptic, antifungal, bacteriostatic, astringent, antioxidant, diastolic, anti-inflammatory, and anesthetic properties [24,25,26]. In this work, we have tested the activity of ethanol extracts of propolis (EEPs) gathered in different regions of Poland against planktonic and biofilm-related cells of *S. aureus*. Additionally, we have investigated the chemical composition and antioxidant potential of collected samples of propolis as well as synergistic interactions of ethanol extracts of propolis (EEPs) with antibacterial antibiotics. The obtained results revealed the high antistaphylococcal potential of Polish propolis. However, important differences in activity and chemical composition of different samples were noted.

## 2. Results

### 2.1. Antibacterial Activity of Ethanol Extracts of Propolis (EEP). Determination of MIC (Minimum Inhibitory Concentration) and MBC (Minimum Bactericidal Concentration) Parameters

The research revealed important differences in the activity of ethanol extracts of Polish propolis against Gram-positive and Gram-negative bacteria (Table 1). Up to the concentration of 4096 µg/mL no activity was observed against both investigated reference strains of Gram-negative bacteria, namely *E. coli* ATCC 25922 and *P. aeruginosa* ATCC 27853. Neither growth inhibition nor bactericidal effect was observed. Much better results were obtained with Staphylococci. However, some important differences were observed between the investigated samples. The highest susceptibility was exhibited by *S. epidermidis* ATCC 12228, with MIC and MBC values in the range of concentrations between 32 µg/mL (three and two products, respectively) and 512 µg/mL (five and six products respectively). Both *S. aureus* strains exhibited slightly higher resistance with MIC values between 128 and 512 µg/mL. However, in contrast to *S. epidermidis*, the bactericidal effect usually required higher concentrations of extracts in comparison to concentrations required for growth inhibition. The same values of MIC and MBC were observed with the eight extracts when tested against *S. aureus* ATCC 29213.

The high anti-staphylococcal potential of propolis collected in Polish apiaries was confirmed for clinical isolates, including 16 methicillin-susceptible strains (MSSA) and five isolates that were methicillin-resistant (MRSA). The investigation was performed for six EEPs. Based on the activity level against reference strains of *S. aureus*, the selected products were classified as: highly active (EEPs 12 and 20); medium active (EEPs 4 and 6) and low active (EEPs 3 and 8). The results presented in Table 2 confirm that EEPs with numbers 12 and 20 exhibited slightly stronger activity against all strains tested, and satisfactory activity was also observed for EEP no. 6. It is important to note that EEPs 12 and 20 effectively eliminated (not only inhibited growth) bacterial cells of all strains tested, with MBC values in the range from 512 to 1024 µg/mL. Other samples, particularly EEP3 and EEP8, but also EEPs 4 and 6, were less effective in the elimination of staphylococcal cells. No differences in the susceptibility were observed between MSSA and MRSA isolates.

### 2.2. Kinetics of the Bactericidal Action of EEPs

**Figure 1:**
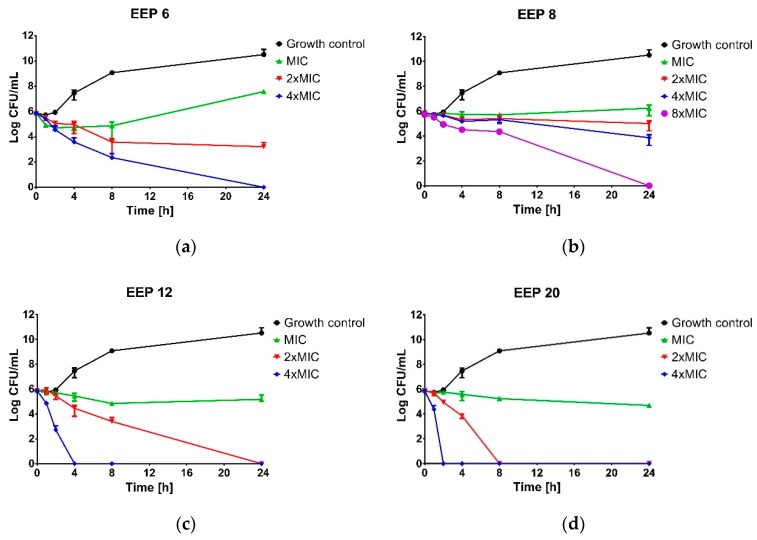
Kill-time assay for selected ethanolic extracts of propolis (EEPs) tested against *S. aureus* ATCC 25923 at or above the MIC. The growth control contained no extracts. (**A**) EEP 6, (**B**) EEP 8, (**C**) EEP 12, (**D**) EEP 20. The results are presented as means ± SD (*n* = 3). Data without error bars indicates that the SD is too small to be observed on the graph.

Important differences in antimicrobial activity of the propolis samples harvested by Polish beekeepers were observed with the time-kill kinetic assay (Figure 1). As expected, all EEPs at MICs resulted in only growth inhibition effect with *S. aureus* ATCC 25923. The same result was observed for EEP6 and EEP8 at a concentration of 2 × MIC and 4 × MIC for EEP8. Even at the concentrations of 4 × MIC and 8 × MIC (for EEP6 and EEP8, respectively) the complete elimination of viable cells of *S. aureus* ATCC 25923 required extended incubation up to 24 h. Higher bactericidal efficiency was observed for EEP12 and EEP20. Both of these EEPs inactivated the bacteria at the concentration of 2 × MIC, however, slightly higher activity was observed with EEP20. With EEP20, the bactericidal effect was achieved after 8 h, while EEP12 required 24 h of incubation. At the concentration of 4 × MIC, complete inactivation of bacteria was achieved after 2 and 4 h for EEP20 and EEP12, respectively.

### 2.3. Combined Action of EEPs with Known Antibacterial Antibiotics

The most active product, EEP20, was selected for analysis of interactions between propolis components and common antibacterial antibiotics. According to the guidelines proposed by Odds (2003), the combination of two antimicrobial agents is considered synergistic when the ΣFIC is ≤0.5, indifferent when the ΣFIC value is between 0.5 and 4.0, and antagonistic when the ΣFIC is ≥4.0 [27]. The results of our study are presented in Table 3. 

The synergistic effect (ΣFICI ≤ 0.5) was observed for five out of 16 investigated agents, namely: amikacin, kanamycin, gentamycin, tetracycline, and fusidic acid. Interestingly, the mode of action of all these antibiotics is inhibition of protein synthesis. Interactions of other agents (*n* = 11) with EEP20 formally have been classified as indifferent (ΣFICI > 0.5). However, some positive interactions of combined treatments (relatively low value of ΣFIC—between 0.5 and 1.0) was observed in the case of the following four inhibitors of translation process: chloramphenicol, erythromycin, mupirocin, linezolid, oxacillin (affects cell wall synthesis), and norfloxacin (affects the process of DNA translation). No antagonistic interaction (ΣFIC ≥ 4) was identified in the study.

### 2.4. The Activity of EEPs Against Staphylococci Growing in the Form of Biofilms

**Figure 2:**
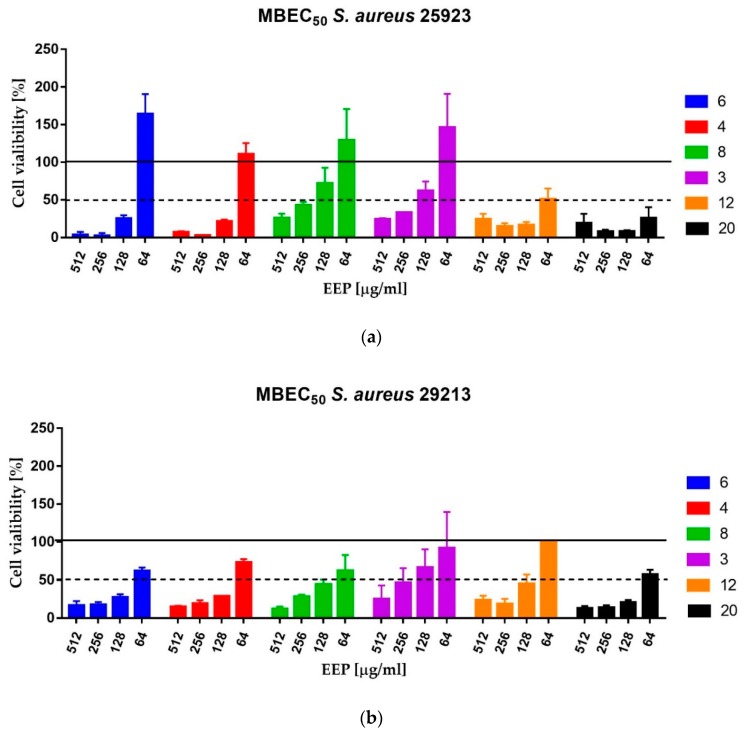
Anti-biofilm activity of selected EEPs against *S. aureus* ATCC 25923 (**A**) and *S. aureus* ATCC 29213 (**B**).

Bacterial biofilms, including *Staphylococcus* spp., exhibit extremely high resistance to most antimicrobial agents, including antibiotics [28,29]. As a consequence, infections caused by staphylococcal biofilm are very difficult for eradication. The investigated extracts of propolis, particularly EEPs 6, 4, 12 and 20 effectively eradicated biofilm of both strains tested using MBEC_50_ values up to 128 µg/mL. In fact, in the case of *S. aureus* ATCC 25923, only residual growth was observed in the medium containing EEPs at concentrations higher than MBEC_50_, and only slightly lower susceptibility was observed for *S. aureus* ATCC 29213. Similarly, with planktonic cells, EEP3 and EEP8 exhibited lower efficacy (Table 1, Table 3 and Figure 2).

### 2.5. Antioxidant Potential and Total Phenolic Content

In addition to the antimicrobial potential, an important advantage of propolis is its’ high antioxidant activity. Herein, we found that all EEPs tested contained a high concentration of polyphenols. The total phenolic content ranged from 96.10 to 197.07 mg GAE/g of the product, with EEP6 having the lowest level and the highest observed with EEP20 (Table 4). The other most active (antimicrobial) product, namely EEP12 also contained a high concentration of polyphenols (195.07 mg GAE/g of the product). However, two other products rich in polyphenols (EEP8 and EEP15) were found as less active against Staphylococci. The EEPs with numbers 12, 20 and 8 also exhibited the highest antioxidant activity in DPPH assay, with IC_50_, µg/mL values of 11.00, 14.55 and 15.10, respectively. The lowest antioxidant potential was identified for EEP6 (IC_50_, µg/mL—75.06). However, the EEP15 (rich in polyphenols) was found to have moderate antioxidant activity (IC_50_, µg/mL—26.65). These results clearly indicate that there is no direct relationship between total phenolic content and antimicrobial or antioxidant activity of EEPs tested.

### 2.6. Determination of Chemical Composition of EEPs with UHPLC-DAD-QqTOF-MS

**Figure 3:**
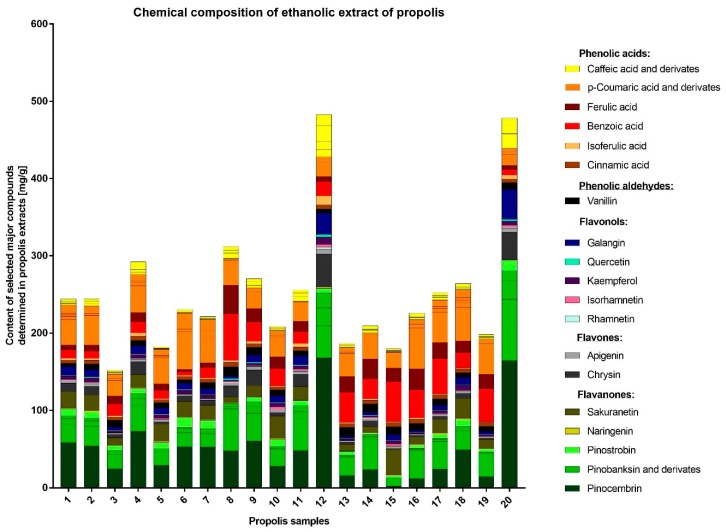
The chemical composition of investigated EEPs.

A total of 69 compounds were identified in the EEP samples by UHPLC-DAD-QqTOF-MS. The chemical profiles of all the samples were characterized by the presence of a large variety of phenolic acids and flavonoids (flavonols, flavones, flavanones) as well as and their esters (mostly of *p*-coumaric and caffeic acids, pinobanksin) (Table 5 and Figure 3). The quantities of the major compounds were determined by UHPLC-DAD and expressed in mg/g of extract. The most abundant compounds were pinocembrin (up to 167.71), pinobanksin-3-*O*-acetate (up to 78.99), benzoic acid (up to 60.26), and *p*-coumaric acid (up to 52.83). Nevertheless, the amount of individual compounds varied greatly between the samples (Table 6). Important differences in concentrations and chemical composition of the polyphenols that were present in two most active (against staphylococci) products EEP12 and EEP20 were noted. In comparison to other investigated EEPs, these two products contained a higher concentration of flavonoids (flavonols, flavones, and flavanones) and lower levels (in percentage) of phenolic acids, especially *p*-coumaric, ferulic, benzoic acids, and their derivatives. EEP12 and EEP20 were especially rich in pinocembrin, pinostrobin, pinobanksin and its derivatives (flavonones), chrysin (flavone) and galangin (flavonol), which appears to be crucial for the high anti-staphylococcal activity of these two propolis samples. Interestingly, EEP8, which was also recognized as a product rich in polyphenols and with high antioxidant activity contained a lower concentration of flavonoids and higher amount of acids which seems not to be very active in the inactivation or inhibition of the growth of bacteria. Thus, the detailed analysis partly explained the observed previously lack of correlation between total phenolic content and antimicrobial/antioxidant activity of the products. However, a significant effort would be required to fully understand the link between antimicrobial/antioxidant potential and chemical composition of propolis, which would be crucial for the prediction of the health-promoting properties of this product.

## 3. Discussion

Since ancient times, propolis, next to herbs, was one of the most important and common agents used for treating infections. The detailed history of using propolis in medicine, as well as discussion of perspectives of its future application, has been recently presented by Silva-Carvalho and coworkers [23]. The results of numerous investigations carried out during the last 40—50 years clearly confirm the high therapeutic (not only antimicrobial) potential of this product. In line with this tendency, we have focused our attention on the antibacterial, primarily anti-staphylococcal, activity of ethanolic extracts of honey bee propolis (EEPs), collected in apiaries located in different regions of Poland. A total of 20 samples were used in the current study. Our previous research revealed the high antifungal potential of Polish propolis [50,51]. The preliminary, screening tests of this study revealed important differences in susceptibility of Gram-positive and Gram-negative bacteria. It is likely a consequence of differences in the cell wall construction of these microorganisms which are also responsible for similar differences of activity of many other agents including antibiotics. In fact, up to the concentration of 4098 µg/mL, no activity was observed against both references strains of Gram-negative pathogenic bacteria: *E. coli* ATCC 25922 and *P. aeruginosa* ATCC 27853. The outcome of this study revealed important differences in the activity of different EEPs, against *S. aureus*, including both reference as well as clinical strains (MIC in the range of concentrations from 32 to 1024 μg/mL).

A similar phenomenon (differences in activity) and comparable level of antistaphylococcal activity have been previously presented by authors who examined the activity of ethanolic extracts of propolis collected in other geographical locations. Brazil is the leader in propolis production and export, with the global market of this product estimated to be approximately 2000 tons [52]. Several independent studies have revealed high efficiency of Brazilian propolis against staphylococci and other important human and animal pathogenic microorganisms. The Regueira group observed high activity of Brazilian red propolis against *S. aureus* (MIC in the range of concentrations from 64 to >1024 µg/mL) but also against Gram-negative bacteria *E. coli* (MIC ranged from 128 to 512 µg/mL) and *P. aeruginosa* (MIC = 512 µg/mL) [53]. Machado and coworkers investigated the antimicrobial potential of three different types of Brazilian propolis: red, brown and green, and ethanolic extracts of the red one were found to be the most active. The MIC of these extracts ranged from 25 to 100 μg/mL for reference strain *S. aureus* ATCC 25923 and from 100 to 400 μg/mL for *S. aureus* ATCC 33591. The ethanolic extracts of other products exhibited a bit lower efficacy with MIC values (for *S. aureus* ATCC 25923) in the ranges 200–800 and 200–400 μg/mL, respectively. The moderate activity against *E. coli* was also observed with MIC values (for ethanolic extracts) from 400 to 1600 μg/mL. The authors also found that ethanolic extracts of all three propolis types exhibited higher antimicrobial potential in comparison to products obtained with supercritical extraction [54]. In regards to both the antimicrobial potential of different types of Brazilian propolis as well as the activity of extracts obtained with ethanol (classical extraction) or carbon dioxide (supercritical extraction), convergent results have been recently published by Devequi-Nunes and colleagues [55]. The Machado group also reported that much higher concentration is required for obtaining the bactericidal effect (MBC) in comparison to MIC [54], which was also observed in our investigation. This conclusion has been additionally supported by the results of kinetic time-kill assay data reported in this study. Even the most active samples, namely 12 and 20, caused only growth inhibition at MIC concentration and at least 2 × MIC was required for inactivation of the indicator strains. This is an important consideration when propolis is considered as an agent for infection treatments.

Interesting results have been published by Suleman and colleagues [56], who investigated 39 propolis samples collected in South Africa. Most of the ethanolic extracts obtained on the base of these products exhibited higher anti-staphylococcal activity in comparison to three control samples of Brazilan propolis, but also in comparison to Brazilian propolis investigated by other authors (presented above). The MIC value for three most active extracts was 6 μg/mL and two of them exhibited bactericidal activity at this concentration [56]. The authors also observed much lower susceptibility of Gram-negative bacteria (for *E.coli* MIC ranged from 391 to 1563 μg/mL) and *C. albicans* (MIC ranged from 98 to 3125 μg/mL), but quite promising activity in the case of another pathogenic yeasts *Cryptococcus neoformans* (MIC between 49 and 391 μg/mL) [56]. Strong antibacterial activities of propolis samples sourced from three different areas of the Sonoran Desert in northwestern Mexico were confirmed in the research of the Velazquez group [57]. The MIC against *S. aureus* of the most active sample (coming from Ures) was 100 μg/mL [57]. The extract of the propolis sample collected near Teheran in Iran inhibited the growth of *S. aureus* ATCC 25923 at a concentration of 250 μg/mL, two times higher concentration was required for bactericidal effect [58]. Similarly, as in our study, some important differences in antibacterial activity were observed within the group of 53 propolis samples collected from different regions of Serbia. The MIC values against *S. aureus* ATCC 25922 ranged from 0.3 mg/mL (one sample, 0.4 mg/mL for nine samples) to 16.1 mg/mL (one sample) [59]. Comparable antimicrobial efficiency against staphylococci was revealed for two propolis samples harvested in subtropical Eastern Australia. MIC values for these samples were 0.4 and 2.0 mg/mL, but in fact, a bactericidal effect was measured in this study [60]. European propolis samples collected from various geographic origins were investigated by Al-Ani and colleagues. Both antimicrobial properties (MIC against Gram-positive microorganisms ranged from 0.08 mg/mL to 2.5 mg/mL) and antioxidant activity of the investigated products were similar to the EEPs tested in our group [61]. Promising, and mostly convergent to our findings, results regarding the anti-staphylococcal activity of ethanolic extract of Polish propolis (EEPP) against methicillin-sensitive *S. aureus* (MSSA) and methicillin-resistant *S. aureus* (MRSA) clinical isolates have been previously published by Wojtyczka and coworkers [62]. However, it should be noted that the authors investigated the propolis from only one location—Kamianna (South Poland). The investigated EEP displayed varying effectiveness against twelve *S. aureus* strains, with MIC in the range from 0.39 to 0.78 mg/mL, determined by broth microdilution method, and minimal bactericidal concentration (MBC) of the EEP ranged from 0.78 to 3.13 mg/mL [62]. The group of Wojtyczka proved also the high activity of Polish propolis against biofilm forming *S. epidermidis* strains [63]. Interesting antimicrobial properties of propolis produced in Polish apiaries was also reported by Scheller and coworkers, who observed synergism between EEP and anti-tuberculosis drugs [64]. For comparison of the therapeutic properties of propolis (including antimicrobial potential), it is important to remember that different methods of extraction of active ingredients are used. Woo and colleagues revealed that using 70% ethanol guaranteed optimal conditions for flavonoids extraction from raw propolis [65]. It is in agreement with the general observation that the highest antimicrobial activity is exhibited with extracts obtained using 70% ethanol, which has been also used herein. Also, the time of extraction differs in procedures applied in different studies. For example, Wojtyczka and co-authors continued extraction for 14 days (versus 100 h proposed herein), which also may be the reason of observed differences in the activity of preparations obtained by these authors and in our study.

From a clinical point of view, the most beneficial aspect of propolis is its anti-biofilm activity. In comparison to planktonic cells, only a slightly lower susceptibility of staphylococci growing in the form of biofilm was observed. The differences were much lower when compared to other antimicrobial agents, e.g., antibiotics. In our opinion, the efficacy of the antibiofilm activity is a result of the multitarget mode of action, in comparison to pure agents (antibiotics), which affect one particular component of a bacterial cell. The promising antibiofilm activity of EEPs produced from raw material collected in other geographical regions, e.g., Russia [66], Turkey [67] and Brazil has been also observed in other studies [68]. Promising results have been also presented by Ambi and coworkers. The authors not only reported high activity of Russian propolis against staphylococcal biofilm but also revealed that removing metals from ethanolic extracts of this product significantly reduces cytotoxicity of this product [69]. Our previous investigation confirmed the high activity of the extract of Polish propolis against candidal biofilms, including high efficiency in biofilm eradication from medical devices such as catheters [51].

Interestingly, our study suggests no differences in susceptibility between MSSA (of different resistance profile against five antibiotics) and MRSA strains. It is in agreement with results presented Pepljnjak and Kosalec, who used diffusion and dilution methods for investigation of the antimicrobial potential of propolis samples collected by Croatian beekeepers [70]. These results suggest that EEPs can be considered as a remedy for the treatment of infections caused by staphylococci resistant to different antibiotics. Propolis is also considered as a component of combined therapy with antibiotics. The outcomes of our study revealed a synergistic effect of EEP20 in combination with five commonly used antibiotics. All the antibiotics that demonstrated synergistic effects with propolis had the same mechanism of action—inhibition of the translation process (at different stages). The convergent results, synergism between EEP and antimicrobial drugs that interfere on bacterial protein synthesis have been published by Fernandez Junior [71]. Onlen and coworkers showed the synergistic effect of the combination of EEP with mupirocin using in vivo animal model—rabbits’ nares infections [72] and Krol and coworkers noted that EEP had a marked synergistic effect on the anti-staphylococcal activity of streptomycin and cloxacillin [73]. Interestingly, the group of Al-Ani observed mostly synergistic effects between EEP and antibiotics acting on cell wall synthesis (vancomycin and oxacillin) [61]. No antagonistic effect between EEP20 and any of the tested antibiotics was observed in our study. Synergistic effect of polish EEP with a broad spectrum of antibiotics of different modes of action was reported by Wojtyczka and colleagues, however, a different method (Kirby and Bauer like) was used by the authors [62]. The results presented in Table 3 indicate that using EEP20 in combination with 6 other antibiotics also resulted in some (slight) positive effect. The MIC values for both agents (EEP20 and tested antibiotic) decreased when they were used together and the ΣFIC values for these combinations were only slightly higher than 0.5, which is currently used as a borderline for classification of an interaction of two agents as synergistic in checkerboard method [27]. However, in regards to the guideline proposed previously by other authors e.g., Eliopoulos and Moellering [74] or and Dimkić and coworkers [75], the interaction of two agents with ΣFIC value in the range between 0.5 and 1.0 was classified as additive. Thus, in our opinion, these positive interactions also could be taken into account for combined therapy with using EEP and antibiotics. Synergistic effects of propolis collected in Poland with important antifungal chemotherapeutics, fluconazole, and voriconazole, was demonstrated in our previous publication [51].

The detailed HPLC analysis revealed the importantly different composition of two samples, namely EEP12 and EEP20, which also exhibited the highest anti-staphylococcal activity. These products were characterized by having a high content of flavonoids. The outcomes of many other studies also suggest that these components are crucial for antimicrobial, antioxidant, or even anticancer potential of propolis. On the other hand, important differences in the chemical composition of this product are an important drawback from the point of view of its medical application. As a consequence, propolis is not commonly used in clinical practice. The specific content of propolis collected in different geographical locations varies to a great extent depending on the plant precursors that are available for bees. On this basis only, 13 different types of Brazilian propolis have been identified and characterized [76]. A recent publication by Isidorov and coworkers have demonstrated that the principal plant precursors of propolis from boreal and temperate zones of the European continent are the bud resins of black poplar (*Populus nigra* L.), downy birch (*Betula pubescens* Ehrh.) and common aspen (*Populus tremula* L.) [31]. These authors have also determined taxonomical markers of the resins harvested from these plant origins [77]. The investigated samples contained a variety of compounds, characteristic for different plant sources, e.g., prenyl esters and some flavonoids characteristic for black poplar, glycerides typical for aspen as well as sakuranetin. The compositions of EEP12 and EEP20 were outstanding. Both samples contained considerably higher amounts of caffeic acid and its derivatives, pinostrobin, pinocembrin, chrysin, galangin and other compounds characteristic for the black poplar. The samples EEP12 and EEP20 contained also higher levels of isoferulic acid, in the case of EEP12 the level of isoferulic acid was two times higher than ferulic acid. Additionally, the samples contained just traces of sakuranetin, similar to the black poplar exudate [31,77]. This suggests that most of the samples originate from both black poplar and aspen. On the other hand, the EEP12 and EEP20 may be classified as being derived from black poplar. Such profiles and differences in composition are also consistent with previous findings [39,78]. It is worth to note that the polyphenol composition of black poplar buds themselves may vary [79]. While ‘typical’ black poplar usually contains more flavonoids than free phenolic acids, the concentration of free phenolic acids is higher in cultivar *P. nigra* ‘Italica’. Its main component is isoferulic acid [31], while other cultivars may contain *p*-coumaric acid as the main free phenolic acid [78].

## 4. Materials and Methods

### 4.1. Chemicals and Drugs

The standard compounds and reagents (all of analytical grade): MTT (3-(4,5-dimethylthiazol-2-yl)-2,5-diphenyltetrazolium bromide), DMSO (dimethyl sulfoxide), gallic acid, Na_2_CO_3_, 1,1-diphenyl-2-picrylhydrazyl radical (DPPH), Folin-Ciocalteu’s reagent, resazurin, tetracycline, chloramphenicol, kanamycin, acetonitrile (LC-MS grade, gradient grade), formic acid, 4-methoxybenzoic acid, 4-hydroxy-3-methoxycinnamaldehyde, caffeic acid, quercetin, luteolin and pinostrobin PBS were purchased from Merck (Darmstadt, Germany). The standards of 4-hydroxybenzoic acid, *p*-coumaric acid, ferulic, isoferulic acid, pinobanksin, chrysin, sakuranetin, naringenin, apigenin, kaempferol, isorhamnetin, acacetin and pinocembrin were obtained from Extrasynthese (Genay, France) and galangin from Alfa Aesar, (Haverhill, MA, USA). The antibiotics: ampicillin, oxacillin, teicoplanin, gentamicin, amikacin, fusidic acid, erythromycin, mupirocin, rifampicin, levofloxacin, norfloxacin, linezolid, bacitracin were purchased from Argenda (Poland). Methanol, ethanol, vanillin, benzoic acid, cinnamic acid were purchased from (POCH, Gliwice, Poland). Ultrapure H_2_O (18.0 MΩ) was obtained with a Milli-Q Advantage A10 system (Millipore, Billerica, MA, USA). The absorbance of the reaction mixture in Folin-Ciocalteu, DPPH assays were measured using a Genesys 20 spectrophotometer (Thermo Scientific, Waltham, MA, USA).

### 4.2. Bacterial Strains and Media

In the preliminary studies, the antimicrobial activity of ethanol extracts of propolis was tested against five reference strains of bacteria: *Staphylococcus aureus* ATCC 25923, *S. aureus* ATCC 29213, *S. epidermidis* ATCC 12228, *Pseudomonas aeruginosa* ATCC 27853, and *Escherichia coli* ATCC 25922. The anti-staphylococcal potential of selected propolis samples was also investigated against sixteen MSSA and five MRSA isolates from patients with different infections (Table 7.). Bacteria were routinely grown on Luria-Bertani Agar (LA, Sigma Aldrich, Schnelldorf, Germany). The Minimum Inhibitory Concentration (MIC) was determined using liquid medium—Mueller-Hinton Broth 2 (MHB2, Sigma Aldrich) and for determination of Minimum Bactericidal Concentrations (MBC) the cells were transferred on the Baird Parker Agar plates (Biomaxima, Lublin, Poland). For biofilm formation TSB liquid medium supplemented with 2.5% of glucose was used.

### 4.3. Preparation of Ethanol Extract of Propolis

**Figure 4:**
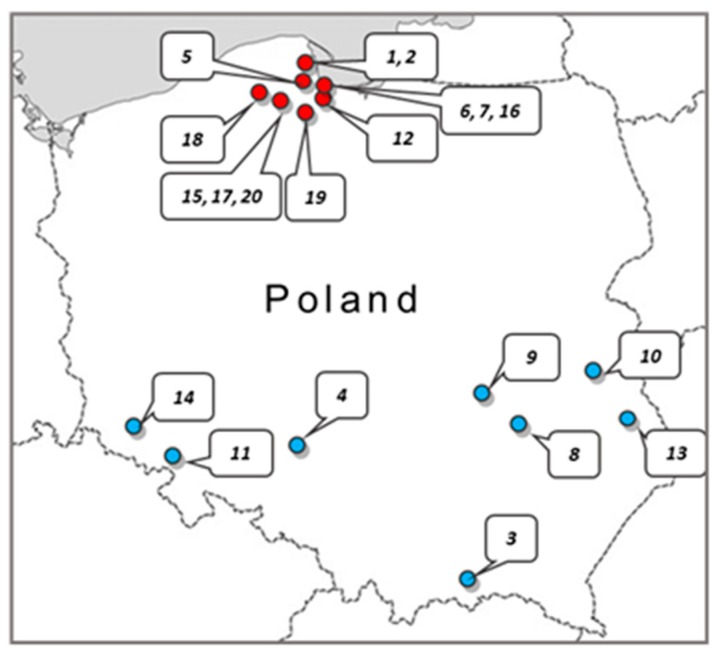
Map of Poland indicating the geographic origin of the propolis samples. The dots (●—Pomerania Province; ●—middle/southern region of Poland) indicate the place where the propolis samples were collected. **1**, **2**—Rumia, 2015; **3**—Kamianna, 2015; **4**—Kluczbork, 2015; **5**—Czeczewo, 2015; **6**, **7**, **16**—Gdansk, 2015; **8** – Lipsko, 2017; **9**—Radom, 2017; **10**—Parczew, 2017; **11**—Walbrzych, 2017; **12**—Pruszcz Gdanski, 2017; **13**—Chelm, 2017; **14**—Debowy Gaj, 2017; **15**, **17**, **20**—Stezyca, 2017; **18**—Jasien, 2017; **19**—Szczodrowo, 2017.

Twenty crude samples of *Apis mellifera* propolis were obtained from apiaries located in the various regions of Poland (Figure 4). The samples were kept under dry storage at ambient temperature in the dark until processing. In all cases, 5 g of raw propolis was extracted with 50 mL of 70% ethanol. Extraction was carried out in the dark for 100 h at ambient temperature with gentle agitation. Afterward, the ethanol extract solutions were centrifuged (9000 rpm) and filtered through Millipore filters (0.22 µm). The filtrates were evaporated to dryness at 40 °C using a rotary vacuum evaporator. The obtained resinous substance was weighed and then the working solutions of the extracts were prepared at a concentration of 81.92 mg/mL in 70% ethanol.

### 4.4. UHPLC-DAD-QqTOF-MS Analysis

UHPLC analyses were performed similarly as described previously [80,81] on Thermo Scientific™ UltiMate™ 3000 system (Thermo Scientific™ Dionex™, Sunnyvale, CA, USA), equipped with an autosampler and DAD detector set at 280, 320 and 360 nm. Spectral data was recorded in the 200–600 nm range. Chromatographic separation was performed on Kinetex^®^ F5 2.6 µm, 100 Å, 150 × 2.1 mm analytical column (Phenomenex, Torrence, CA, USA) thermostated at 35 ± 1°C. Injection volume was set to 1 μL. The mobile phase consisted of 0.1% formic acid solutions in water (solvent A) and acetonitrile (solvent B). The flow rate was set at 0.4 mL/min and the separation was obtained using gradient: 100% of solvent A, decreasing to 91% within 7 min, isocratic for 3 min, decreasing to reach 80% in minute 10.5 and 60% in minute 18.5, isocratic for another 4 min and decreasing to reach 0% in minute 28.5, isocratic until 32 min. Subsequently, it returned to 100% A and the system was stabilized before the next analysis.

UHPLC-DAD-QqTOF-MS was performed in a similar setting and chromatographic conditions, additionally using Compact QqTOF-MS detector (Bruker, Darmstadt, Germany). MS detector was used in ESI negative mode, ion source temperature was set at 100 °C, nebulizer gas pressure was set at 2.0 bar, dry gas flow 0.8 L/min and temperature 210 °C. The capillary voltage was set at 2.20 kV. The collision energy was set at 8.0 eV. Internal calibration was obtained using 10 mM solution of sodium formate clusters. For ESI-MS/MS experiments, collision energy was set at 35 eV and nitrogen was used as collision gas.

Before the analysis, all the extracts were dissolved in ethanol and filtered through CHROMAFIL^®^ 0.2 µm, Ø13mm, H-PTFE membrane syringe filter (Macherey-Nagel, Düren, Germany). Standard compounds were dissolved in ethanol or DMSO-ethanol mixture (1:10) for hardly soluble flavonoids and subsequently diluted in order to obtain calibration curves in the range of concentrations 0.5–200 µg/mL. Amounts of different compounds in the samples were calculated based on the calibration curve of appropriate standard or corresponding parent compound (e.g., amount of pinobanksin-3-*O*-acetate was expressed as pinobanksin equivalents).

### 4.5. Measurement of DPPH Radical Scavenging

The antiradical potential of the EEP was measured using DPPH radicals. The 2-fold serial dilutions of EEPs ranging from 0.008 mg/mL to 1.0 mg/mL were prepared in methanol in 96-well microtiter plates. Afterward, the 0.2 mM solution of DPPH was added to the plate at the 1:1 proportion. The mixture was incubated for 30 min in dark, at ambient temperature. Then, the absorbance was measured at 515 nm against a blank using a microplate reader. DPPH solution and methanol was used as a control, while ascorbic acid was used as a reference in comparing to the propolis extract. All measurements were performed in triplicate. The data were expressed as the percentage of DPPH reduction and calculated using the formula below. IC_50_ values were calculated using GraphPad Prism^®^ 5 (Version 5.01, GraphPad Software, Inc., La Jolla, CA, USA):

$$\mathrm{Antioxidant}\;\mathrm{activity}\;\left(\%\right)=\frac{100\;\times \;[A515\;\left(\mathrm{radical}\right)\;\times \;A515\;\left(\mathrm{sample}\right)}{A515\;\left(\mathrm{radical}\right)}$$

### 4.6. Determination of Total Phenolic Content (TPC) of Propolis

The total content of phenolic compounds was determined using a Folin-Ciocalteu method as described before [82]. The Folin-Ciocalteu reagent in the volume of 0.5 mL was mixed with 100 μL of the EEPs (5 µg/mL in ultrapure water) and after 5 min, 3 mL of 100 g/L solution of Na_2_CO_3_ (*w*/*v*) was added. Following shaking the mixture was made up to a volume of 10 mL with ultrapure water and incubated for 90 min at ambient temperature. The absorbance at 720 nm was measured against blank in a 10 mm quartz cuvette. Total phenol content was calculated and expressed as milligrams of gallic acid equivalent (GAE) per kilogram, using a calibration curve prepared with a fresh gallic acid standard solution (10–2000 mg/L). All measurements were performed in triplicate.

### 4.7. Investigation of Antimicrobial Potential of Propolis

The minimum inhibitory concentrations (MICs) were determined by the two-fold broth microdilution method according to the CLSI standard methodology [83]. Bacteria were plated on LB solid medium and incubated overnight at 37 °C. Two to three bacterial colonies were taken directly from the plate and transferred into PBS buffer (pH = 7.4). The bacterial suspension was adjusted to the optical density of OD_600_ = 0.1 and diluted in MHB2 medium at a ratio of 1:100 *v/v* to the final cell concentration of approximately 1.0 × 10^6^ CFU/mL. Two-fold serial dilutions of the propolis were prepared with a final dilution rate of 4096, 2048, 1024, 512, 256, 128, 64, 32, 16, 8 μg/mL. An aliquot of 100 μL of inoculum was dispensed to the wells of columns 1–11. Column 11 contained 200 µL of inoculum, and column 12 contained 200 µL of the MHB2 broth only (as control of sterility). The plates were incubated 24 h under static conditions at 37 °C. Because color and solubility of propolis extracts may interfere with growth measurement, the resazurin test was used. After incubation, resazurin (0.015% in PBS buffer) was added to all wells (30 µL), and further incubated for 2 h at 37 °C in the dark. The lowest concentration with no color change (blue resazurin color remained unchanged) was taken as a MIC value. The minimum bactericidal concentrations (MBCs) were assessed by transferring each dilution used for MIC assay on Baird-Parker agar plates using a sterile 48-well microtiter plate replicator. The plates were incubated for 24 h at 37 °C. Concentrations, where no growth of the colonies was observed, were assigned as MBC.

### 4.8. Kill-Time Assay

Time-kill assay was performed for four propolis samples revealed the highest (EEP20 and EEP12), moderate (EEP6) and weak (EEP8) anti-staphylococcal activity. The suspension of approx. cell density 1.0 × 10^6^ CFU/mL of *S. aureus* ATCC 25923 was prepared in MHB2 broth supplemented with ethanolic extracts of propolis to the final concentrations equal to MIC, 2 × MIC, 4 × MIC or 8 × MIC and incubated at 37 °C with shaking. Bacterial suspension without propolis addition was used as the untreated control. At predetermined time intervals (0, 1, 2, 4, 6, 8 and 24 h) samples were taken, serially diluted in PBS buffer (from 10^−1^ to 10^−7^) and spotted (10 µL) onto a Baird-Parker agar plate. After 24 h of incubation, the plates were enumerated. Bactericidal activity was determined if a greater then 3 log10-fold decrease in the number of survivors was noted.

### 4.9. Biofilm Formation in 96-Well Microtiter Plate

The assay for biofilm cultivation was performed according to the procedure described previously with slight modifications [84]. The suspensions of approx. cell density 1-5 × 10^8^ CFU/mL of *S. aureus* ATCC 25923 and *S. aureus* ATCC 29213 were diluted 1:100 (*v*/*v*) in TSB medium supplemented with 2.5% of glucose. Two hundred microliters of the cell suspensions were placed into the wells of columns 1–7 of vertically set plates. Negative controls were performed with a sterile medium placed in the wells of column 8. The plates were incubated for 24 h at 37 °C without shaking in order to allow bacteria to attach.

### 4.10. MBEC Assay

The Minimum Biofilm Eradication Concentration (MBEC) assay was carried out as described previously. The medium was removed and the biofilm was gently washed with 200 μL of sterile PBS. The 2-fold serial dilutions of propolis in MHB2 medium ranging from 2048 to 128 µg/mL were added to the wells and incubated for 24 h at 37 °C. The assay was performed for six propolis samples: EEP12 and EEP20—the most active against planktonic cells, EEP4 and EEP6—classified as samples of medium activity against planktonic cells and two samples that exhibited low activity—EEP3 and EEP8. The MBEC_50_ values were taken as the lowest concentration of propolis that caused eradication of at least 50% of living cells in comparison to the cells growing in the untreated control—measured as comparison of ability of living cells to the biotransformation of MTT (3-(4,5-dimethyl-2-thiazolyl)-2,5-diphenyl-2*H*-tetrazolium bromide) to insoluble in water violet formazan crystals [85]. The MTT assay was performed as described previously [85,86]. Briefly, after the biofilm formation (according to the procedure presented above) the inoculum was removed and the wells of microplate were washed with 200 μL of sterile PBS buffer. Subsequently, 150 μL of PBS and 50 μL of MTT solution (0.3% in PBS) were added to the wells and mixed. Following 2 h incubation at 37 °C in the dark, the MTT solution was replaced with 200 μL of DMSO for dissolving of formed formazan crystals. The optical density of the obtained solutions was measured at 540 nm using a Victor^3^ microtiter reader (Perkin Elmer, Waltham, MA, USA).

### 4.11. Checkerboard Dilution Test

The checkerboard test was used to evaluate the antimicrobial effect of propolis and different antibiotics in combination. Two samples of propolis (EEP12 and EEP20) with the highest antimicrobial activity and sixteen antibiotics were tested. Two-fold dilutions of propolis extracts were prepared along the ordinate in a 96-well plate. Then, previously prepared two-fold dilutions of antibiotic were distributed along the abscissa. Final concentrations of both antibiotic and propolis range from at least MIC to 1/32 MIC. Afterward, each microtiter well was inoculated with a bacterial inoculum of 1.0 × 10^6^ CFU/mL in a volume equal to the volume of diluted antimicrobial solution (final cell concentration in each well was 5.0 × 10^5^ CFU/mL), and the plates were incubated at 37 °C for 24 h under static conditions. After incubation, resazurin (0.015% in PBS buffer) was added to all wells (30 µL), and further incubated for 2 h at 37 °C in the dark. The change of color from blue to pink indicated the growth of bacteria. Fractional inhibitory concentration index (ΣFIC) was calculated for each well as follow:

$$\sum FIC=\frac{MIC\;of\;EEP\;in\;combination\;with\;antibiotic}{MIC\;of\;EEP\;alone}+\frac{MIC\;of\;antibiotic\;in\;combination\;with\;EEP}{MIC\;of\;antibiotic\;alone}$$

ΣFICs were interpreter according to the model suggested by Odds [27]. A synergistic effect was observed when ΣFIC ≤ 0.5; an indifferent effect when 0.5 < ΣFIC < 4 and an antagonistic effect when ΣFIC ≥ 4.

### 4.12. Data Analysis

All experiments in this study were completed in triplicate and data was expressed as the means ± SD.

## 5. Conclusions

Our research revealed that propolis produced in Polish apiaries exhibits considerable anti-staphylococcal activity. The ethanolic extracts of this natural product effectively eliminate biofilm as well as planktonic cells of *S. aureus*. However, some important differences in the antimicrobial efficiency of the products were observed. The HPLC analysis revealed that two products that exhibited the highest antibacterial potential were characterized by having a high content of flavonoids, which suggests that these compounds are crucial for antimicrobial activity of Polish propolis. From a clinical point of view especially important was observed the synergistic effect of EEPs with antibiotics that affect protein synthesis in bacterial cells. Another, important health beneficial property of investigated samples of propolis was high antioxidant potential.

## Figures and Tables

**Table 1:** Antibacterial activity of ethanol extracts of propolis (EEPs) collected from apiaries located in different regions of Poland tested against reference strains of bacteria.

No. EEP.	MIC and MBC (µg/mL) Against Different Strains of Bacteria									
	*S. aureus* ATCC 25923		*S. aureus* ATCC 29213		*S. epidermidis* ATCC 12228		*E. coli* ATCC 25922		*P. aeruginosa* ATCC 27853	
	MIC	MBC	MIC	MBC	MIC	MBC	MIC	MBC	MIC	MBC
1	256	2084	256	256	64	64	>4096	>4096	>4096	>4096
2	256	2084	128	1024	32	64	>4096	>4096	>4096	>4096
3	512	4096	512	4096	512	512	>4096	>4096	>4096	>4096
4	256	4096	256	256	64	64	>4096	>4096	>4096	>4096
5	256	2084	256	2048	64	128	>4096	>4096	>4096	>4096
6	256	1024	256	256	128	128	>4096	>4096	>4096	>4096
7	256	2048	256	4096	128	256	>4096	>4096	>4096	>4096
8	512	>4096	512	4096	512	512	>4096	>4096	>4096	>4096
9	256	4096	256	1024	64	64	>4096	>4096	>4096	>4096
10	256	4096	256	4096	64	64	>4096	>4096	>4096	>4096
11	256	512	256	512	32	32	>4096	>4096	>4096	>4096
12	128	512	128	128	64	64	>4096	>4096	>4096	>4096
13	512	4096	512	512	512	512	>4096	>4096	>4096	>4096
14	512	2048	512	512	256	256	>4096	>4096	>4096	>4096
15	512	>4096	512	1024	256	512	>4096	>4096	>4096	>4096
16	512	2048	256	512	512	512	>4096	>4096	>4096	>4096
17	256	2048	256	512	256	256	>4096	>4096	>4096	>4096
18	256	512	256	256	128	128	>4096	>4096	>4096	>4096
19	512	1024	512	512	512	512	>4096	>4096	>4096	>4096
20	128	256	128	128	32	32	>4096	>4096	>4096	>4096

**Table 2:** Antibacterial activity of selected ethanolic extracts of propolis collected from Polish apiaries against clinical isolates of *S. aureus* (MSSA and MRSA).

Strain No.	MIC and MBC (µg/mL) against Clinical Isolates of *S. aureus*											
	EEP 4		EEP 3		EEP 6		EEP 8		EEP 12		EEP 20	
	MIC	MBC	MIC	MBC	MIC	MBC	MIC	MBC	MIC	MBC	MIC	MBC
1	128	2048	512	>2048	128	2048	256	>2048	256	512	128	512
2	256	>2048	512	>2048	128	>2048	512	>2048	128	1024	256	1024
3	256	>2048	512	>2048	256	>2048	256	>2048	128	1042	128	1024
4	256	>2048	512	>2048	128	2048	512	>2048	256	512	256	512
5	256	>2048	256	>2048	128	>2048	512	>2048	128	512	128	512
6	256	>2048	512	>2048	128	1024	256	>2048	128	1024	128	512
7	128	>2048	512	>2048	128	2048	512	>2048	256	1024	128	512
8	256	>2048	512	>2048	128	512	512	>2048	256	1024	128	512
9	256	>2048	512	>2048	128	1024	512	>2048	128	512	128	512
10	64	1024	512	>2048	512	1024	32	>2048	32	1024	64	1024
11	256	1024	512	>2048	256	1024	512	>2048	128	512	128	512
12	256	1024	512	>2048	128	2048	512	>2048	128	512	128	1024
13	256	2048	512	>2048	128	1024	512	>2048	128	512	128	512
14	256	2048	512	>2048	128	1024	512	>2048	256	512	128	512
15	256	>2048	512	>2048	256	512	512	>2048	128	1024	128	1024
16	256	2048	512	>2048	512	1024	128	>2048	128	512	256	512
17 *	256	1024	512	2048	128	2048	512	>2048	256	512	128	512
18 *	128	1024	256	>2048	64	512	256	>2048	128	512	128	512
19 *	256	512	512	>2048	128	512	512	>2048	128	512	128	512
20 *	512	1024	512	>2048	256	512	512	>2048	256	512	256	512
21 *	256	512	1024	>2048	256	512	1024	>2048	256	512	128	512

* Methicillin-resistant.

**Table 3:** The results of checkerboard analysis of drug interaction between EEP20 and different antibiotics against *S. aureus* ATCC 25923.

Agent	MIC (µg/mL)		FIC	ΣFIC	Interpretation
	Alone	Combination			
EEP 20	128	16	0.125	0.375	Synergy
Amikacin	1	0.25	0.25		
EEP 20	128	32	0.25	0.5	Synergy
Kanamycin	2	0.5	0.25		
EEP 20	128	32	0.25	0.5	Synergy
Gentamicin	0.125	0.0312	0.25		
EEP 20	128	16	0.125	0.375	Synergy
Tetracycline	0.5	0.125	0.25		
EEP 20	128	16	0.125	0.625	Indifferent *
Chloramphenicol	4	2	0.5		
EEP 20	128	16	0.125	0.375	Synergy
Fusidic acid	0.5	0.125	0.25		
EEP 20	128	16	0.125	0.625	Indifferent *
Erythromycin	0.5	0.25	0.5		
EEP 20	128	16	0.125	0.625	Indifferent *
Linezolid	4	2	0.5		
EEP 20	128	128	1	2	Indifferent
Levofloxacin	0.125	0.125	1		
EEP 20	128	32	0.25	0.750	Indifferent *
Norfloxacin	0.5	0.25	0.5		
EEP 20	128	128	1	2	Indifferent
Rifampicin	0.015	0.015	1		
EEP 20	128	32	0.25	0.750	Indifferent *
Mupirocin	0.25	0.125	0.5		
EEP 20	128	64	0.5	1	Indifferent
Bacitracin	32	16	0.5		
EEP 20	128	64	0.5	0.625	Indifferent *
Oxacillin	0.25	0.062	0.125		
EEP 20	128	64	0.5	1	Indifferent
Ampicillin	0.25	0.125	0.5		
EEP 20	128	64	0.5	1	Indifferent
Teicoplanin	2	1	0.5		

Indifferent *—according to the classification proposed by Odds combined therapy with these values of ΣFIC should be classified as indifferent, however, some slight decrease of MIC values of both agents (used in combination) was observed.

**Table 4:** Antioxidant activity of EEPs and total phenolic content.

EEP’s Number	DPPH [IC_50_, µg/mL]	Total Phenolic Content [mg GAE/g]
1	31.49	122.34 ± 5.9
2	27.86	159.84 ± 4.9
3	23.63	107.7 ± 5.6
4	39.80	132.01 ± 3.6
5	45.62	130.94 ± 0.4
6	75.06	96.10 ± 2.2
7	56.49	128.88 ± 3.6
8	15.10	182.28 ± 7.1
9	22.63	157.01 ± 5.0
10	27.45	131.13 ± 7.1
11	21.29	158.71 ± 4.3
12	11.00	195.07 ± 3.2
13	26.94	130.07 ± 0.9
14	24.08	157.16 ± 8.2
15	25.65	182.88 ± 2.4
16	24.07	151.19 ± 5.2
17	23.52	144.22 ± 2.5
18	31.28	132.68 ± 2.1
19	25.95	140.59 ± 8.2
20	14.59	197.07 ± 3.0

**Table 5:** Compounds identified in propolis samples by UHPLC-DAD-QTOF-MS.

No.	Component	RT [min]	UV max [nm]	[M − H^+^]^−^	Reference	Sample Number																			
						1	2	3	4	5	6	7	8	9	10	11	12	13	14	15	16	17	18	19	20
1	4-Hydroxybenzoic acid ^a,b,c^	7.32	**256**	137.0248	[30]	+	+	+	+	+	+	+	+	+	+	+	+	+	+	+	+	+	+	+	+
2	3-Hydroxybenzoic acid ^b,c^	8.00	296, **233**	137.0245	[30]	+	+	+	+	+	+	+	+	+	+	+	+	+	+	+	+	+	+	+	+
3	Caffeic acid ^a,b,c^	11.02	**323**, 295sh	179.0351	[31,32,33,34,35,36,37,38]	+	+	+	+	+	+	+	+	+	+	+	+	+	+	+	+	+	+	+	+
4	Vanillin ^a,b,c^	12.41	**310**, 280, 230	151.0404	[36,38]	+	+	+	+	+	+	+	+	+	+	+	+	+	+	+	+	+	+	+	+
5	Benzoic acid ^a,b,c^	13.46	274, **230**	121.0296	[36,38]	+	+	+	+	+	+	+	+	+	+	+	+	+	+	+	+	+	+	+	+
6	*p*-Coumaric acid ^a,b,c^	13.81	**310**, 300sh	163.0401	[31,32,33,34,35,36,37,38]	+	+	+	+	+	+	+	+	+	+	+	+	+	+	+	+	+	+	+	+
7	Ferulic acid ^a,b,c^	14.63	**322**, 298sh	193.0497	[31,32,33,34,36,37]	+	+	+	+	+	+	+	+	+	+	+	+	+	+	+	+	+	+	+	+
8	Isoferulic acid ^a,b,c^	14.74	**324**, 300sh	193.0497	[31,32,33,36,37,38]	+	+	+	+	+	+	+	+	+	+	+	+	+	+	+	+	+	+	+	+
9	4-Methoxybenzoic acid ^a,b,c^	15.06	**256**	nd	[30]	+	+	+	+	+	+	+	+	+	+	+	+	+	+	+	+	+	+	+	+
10	4-Hydroxy-3-methoxycinnamaldehyde ^a,b,c^	15.39	**339**, 301sh	177.0554	[30]	+	+	+	+	+	tr	+	+	+	+	+	+	+	+	+	+	+	+	+	tr
11	Dimethylcaffeic acid (DMCA) ^b,c^	16.40	**324**, 294sh	207.0659	[33,37]	+	+	+	+	+	+	+	+	+	+	+	+	+	+	+	+	+	+	+	+
12	Cinnamic acid ^a,b,c^	16.75	**278**	147.0451	[33,36,37,39]	+	+	+	+	+	+	+	+	+	+	+	+	+	+	+	+	+	+	+	+
13	Caffeic acid ethyl ester	17.29	**322**, 298	207.0670	[40]	+	+	+	+	+	+	+	+	+	+	+	+	+	+	+	+	+	+	+	+
14	Pinobanksin-5-methylether ^b,c^	17.32	**288**	285.0762	[32,33,35,37]	+	+	+	+	+	+	+	+	+	+	+	+	+	+	+	+	+	+	+	+
15	Quercetin ^a,b,c^	17.89	364, 270sh, **265**	301.0349	[33,36,37,39]	+	+	+	+	+	+	+	+	+	+	+	+	+	+	+	+	+	+	+	+
16	Luteolin ^a,b,c^	17.94	**345**, 254	285.0412	[30,39]	+	+	+	+	+	+	+	+	+	+	+	+	+	+	+	+	+	+	+	+
17	Pinobanksin ^a,b,c^	18.44	**292**	271.0611	[31,32,33,37]	+	+	+	+	+	+	+	+	+	+	+	+	+	+	tr	+	+	+	+	+
18	Quercetin-3-methyl ether ^b,c^	18.45	355, 268sh, **255**	315.0497	[33]	tr	tr	tr	tr	tr	tr	tr	tr	tr	tr	tr	tr	tr	tr	tr	tr	tr	tr	tr	tr
19	Chrysin-5-methyl ether ^b,c^	18.70	314sh, **264**	267.0663	[35,41]	+	tr	tr	tr	–	tr	tr	tr	+	tr	+	+	tr	tr	–	–	tr	tr	tr	+
20	Naringenin ^a,b,c^	18.92	**289**	271.0612	[37,41]	+	+	+	+	+	+	+	+	+	+	+	+	+	+	+	+	+	+	+	+
21	Apigenin ^a,b,c^	19.26	**338**, 290sh, 263	269.0450	[33,37,39]	+	+	+	+	+	+	+	+	+	+	+	+	+	+	+	+	+	+	+	+
22	Kaempferol ^a,b,c^	19.44	**366**, 295sh, 265	285.0403	[31,36,37,39]	+	+	+	+	+	+	+	+	+	+	+	+	+	+	+	+	+	+	+	+
23	1,3-Di-*p*-coumaroylglycerol ** ^b,c^	19.57	**312**, 300sh	383.1129	[31]	+	+	+	+	+	+	+	+	+	+	+	+	+	+	+	+	+	+	+	+
24	Isorahmnetin ^a,b,c^	19.72	371, 268sh, **256**	315.0502	[31,32,36,37,39,41]	+	+	+	+	+	+	+	+	+	+	+	+	+	+	+	+	+	+	+	+
25	*p*-Coumaroyl-feruloylglycerol ^b,c^	19.85	**316**, 298sh	413.1240	[37]	+	+	+	+	+	+	+	+	+	+	+	+	+	+	+	+	+	+	+	+
26	2-Acetyl-1,3-di-caffeoylglycerol ^b,c^	19.92	**328**, 298sh	457.1470	[34,37,42]	+	+	+	+	+	-	+	+	+	+	+	+	+	+	+	+	+	+	+	+
27	Luteolin-5-methyl ether ^b,c^	20.06	350, 298sh, **267**	299.0549	[33]	+	+	+	+	+	+	+	+	+	+	+	+	+	+	+	+	+	+	+	+
28	Galangin-5-methyl ether ^b,c^	20.26	352, 300sh, **260**	283.0602	[32,33]	+	+	+	+	+	tr	tr	tr	+	+	+	+	+	+	tr	tr	+	tr	+	+
29	Quercetin-3,3′-dimethyl ether ^b,c^	20.36	356, 269sh, **255**	329.0651	[33,37]	+	+	+	+	+	+	+	+	+	+	+	+	+	+	+	+	+	+	+	+
30	Caffeic acid butyl or isobutyl ester ^b, c^	20.73	**326**, 298sh	235.0972	[31,43]	+	+	+	+	+	+	+	+	+	+	+	+	+	+	+	+	+	+	+	+
31	Rhamnetin (quercetin-7-methyl ether) ^b,c^	20.91	356, 268sh, **256**	315.0504	[37,39,44]	+	+	+	+	+	+	+	+	+	+	+	+	+	+	+	+	+	+	+	+
32	Caffeic acid prenyl or isoprenyl ester I ^b,c^	21.04	**325**, 298sh	247.0979	[32,33,35,36,37]	+	+	+	+	+	+	+	+	+	+	+	+	+	+	+	+	+	+	+	+
33	2-Acetyl-1-caffeoyl-3-*p*-coumaroylglycerol ^b,c^	21.22	**316**, 299sh	441.1182	[31,34,37,42]	+	+	+	+	+	+	+	+	+	+	+	+	+	+	+	+	+	+	+	+
34	Caffeic acid prenyl or isoprenyl ester II ^b,c^	21.23	**324**, 298sh	247.0976	[32,33,35,36,37]	+	+	+	+	+	+	+	+	+	+	+	+	+	+	+	+	+	+	+	+
35	Caffeic acid prenyl or isoprenyl ester III ^b,c^	21.33	**325**, 298sh	247.0973	[32,33,35,36,37]	+	+	+	+	+	+	+	+	+	+	+	+	+	+	+	+	+	+	+	+
36	2-Acetyl-1-caffeoyl-3-feruloylglycerol ^b,c^	21.50	**322**, 300sh	471.1300	[34,42]	+	+	+	+	+	+	+	+	+	+	+	+	+	+	+	+	+	+	+	+
37	Caffeic acid benzyl ester ^b,c^	21.64	**328**, 298sh	269.0818	[32,33,37]	+	+	+	+	+	+	+	+	+	+	+	+	+	+	+	+	+	+	+	+
38	Quercetin-3,7-dimethyl ether ^b,c^	21.65	356, 268sh, **256**	329.0659	[30,37]	+	+	+	+	+	+	+	+	+	+	+	+	+	+	+	+	+	+	+	+
39	Chrysin ^a,b,c^	21.93	312sh, **268**	253.0505	[31,33,34,35,36,37]	+	+	+	+	+	+	+	+	+	+	+	+	+	+	+	+	+	+	+	+
40	Pinocembrin ^a,b,c^	22.12	**290**	255.0666	[33,34,35,36,37]	+	+	+	+	+	+	+	+	+	+	+	+	+	+	+	+	+	+	+	+
41	Caffeic acid pentyl or isopentyl ester ^b,c^	22.25	**326**, 298sh	249.1141	[43,45]	tr	tr	tr	tr	tr	tr	tr	tr	tr	tr	tr	tr	tr	tr	–	tr	tr	tr	tr	+
42	Sakuranetin ^b,c^	22.38	**290**	285.0773	[36,37,41]	+	+	+	+	+	+	+	+	+	+	+	tr	+	+	+	+	+	+	+	tr
43	Caffeic acid phenethyl ester (CAPE) ^b,c^	22.40	**325,** 295	283.0986	[32,33,37]	+	+	+	+	+	+	+	+	+	+	+	+	+	+	+	+	+	+	+	+
44	Galangin ^a,b,c^	22.43	360, **266**,	269.0454	[30,31,32,33,35,37]	+	+	+	+	+	+	+	+	+	+	+	+	+	+	+	+	+	+	+	+
45	Acacetin (Apigenin-4′-methyl ether) ^a,b,c^	22.48	**335,** 299sh, 268	283.0621	[36,37,41]	tr	tr	tr	tr	tr	tr	tr	tr	tr	tr	tr	tr	tr	tr	tr	tr	tr	tr	tr	tr
46	2-Acetyl-1,3-di-*p*-coumaroylglycerol ** ^b,c^	22.72	**312,** 300	425.1232	[31,37,42]	+	+	+	+	+	+	+	+	+	+	+	+	+	+	+	+	+	+	+	+
47	Pinobanksin 3-*O*-acetate ^b,c^	22.80	**295**	313.0713	[31,32,33,37]	+	+	+	+	+	+	+	+	+	+	+	+	+	+	+	+	+	+	+	+
48	Kaempferide (kaempferol-4′-methyl ether) ^b,c^	22.93	**365**, 267	299.0555	[30,36]	+	+	+	+	+	+	+	+	+	+	+	+	+	+	+	+	+	+	+	+
49	*p*-Coumaric acid prenyl or isoprenyl ester I ^b,c^	23.11	**311**, 299sh	231.1028	[32,33,35]	+	tr	tr	tr	tr	tr	tr	tr	+	tr	+	+	tr	tr	tr	–	tr	tr	tr	+
50	2-Acetyl-3-*p*-coumaroyl-1-feruloylglycerol ** ^b,c^	23.12	**318**, 299sh	455.134	[31,34]	+	+	+	+	+	+	+	+	+	+	+	+	+	+	+	+	+	+	+	+
51	Methoxychrysin ^b,c^	23.21	310sh, **266,** 245sh	283.0611	[32,33,46]	+	+	+	+	+	+	+	+	+	+	+	+	+	+	+	+	+	+	+	+
52	*p*-Coumaric acid prenyl or isoprenyl ester II ^b,c^	23.38	**310**, 299sh	231.1025	[32,33,35]	+	+	+	+	+	+	+	+	+	+	+	+	+	+	+	+	+	+	+	+
53	*p*-Coumaric acid prenyl or isoprenyl ester III ^b,c^	23.52	**311**, 299sh	231.1028	[32,33,35]	+	+	+	+	+	+	+	+	+	+	+	+	+	+	+	+	+	+	+	+
54	2-Acetyl-1,3-di-feruloylglycerol ^b,c^	23.62	**328**, 298sh	485.1423	[31,34,47]	tr	tr	tr	tr	+	tr	+	+	+	+	+	+	+	+	+	+	+	tr	+	tr
55	*p*-Coumaric acid benzyl ester ^b,c^	23.88	**312**, 298sh	253.0870	[31,32,33]	+	+	+	+	+	+	+	+	+	+	+	+	+	+	+	+	+	+	+	+
56	Caffeic acid cinnamyl ester ^b,c^	24.32	**326**, 300sh	295.0971	[33,37,46]	+	+	+	+	+	+	+	+	+	+	+	+	+	+	tr	+	+	+	+	+
57	Ferulic acid benzyl ester * ^b,c^	24.62	**326**, 298	283.0968	[31,38,48]	+	+	+	+	+	+	+	+	+	+	+	+	+	+	+	+	+	+	+	+
58	Pinobanksin 3-*O*-propanoate ^b,c^	25.05	**294**	327.0876	[32,33,37]	+	+	+	+	–	–	tr	+	+	+	+	+	+	+	+	–	+	tr	tr	+
59	*p*-Coumaric acid phenethyl ester ^b,c^	25.06	**310**, 300sh	267.1033	[35]	+	+	+	+	+	+	+	+	+	+	+	+	+	+	+	+	+	+	+	+
60	*p*-Coumaric acid pentyl or isopentyl ester I ^b,c^	25.16	**310**, 298sh	233.1190	[43]	+	+	+	+	+	+	+	+	+	+	+	+	+	tr	+	+	+	+	+	+
61	*p*-Coumaric acid pentyl or isopentyl ester II ^b,c^	25.26	**311**, 298sh	233.1192	[43]	+	+	+	+	+	+	+	tr	+	+	+	+	+	+	tr	+	+	+	+	+
62	*p*-Coumaric acid cinnamyl ester ^b,c^	26.83	**312**, 300sh	279.1024	[37]	+	+	+	+	+	+	+	+	+	+	+	+	+	+	+	+	+	+	+	+
63	Pinobanksin 3-*O*-butanoate or isobutanoate ^b,c^	26.92	**293**	341.1022	[32,33]	+	+	+	+	tr	tr	+	+	+	+	+	+	+	+	tr	tr	tr	tr	tr	tr
64	Pinostrobin chalcone ^b,c^	26.93	**339,** 287sh	269.0823	[37]	+	+	+	+	+	+	+	+	+	+	+	+	+	+	+	+	+	+	+	+
65	Pinobanksin 3-*O*-pentenoate or isopentenoate ^b,c^	27.06	**295**	353.1025	[37,44,49]	+	+	+	+	–	tr	+	+	+	+	+	+	+	+	tr	tr	+	tr	+	+
66	Pinostrobin (pinocembrin-7-methyl ether) ^a,b,c^	27.20	**289**	269.0811	[37]	+	+	+	+	+	+	+	+	+	+	+	+	+	+	+	+	+	+	+	+
67	Pinobanksin 3-*O*-pentanoate or isopentanoate ^b,c^	27.69	**292**	355.1188	[32,33,37]	+	+	+	+	tr	tr	+	+	+	+	+	+	+	+	tr	tr	+	+	+	+
68	Metoxycinnamic acid cinnamyl ester ^b,c^	27.74	**280**	293.2122	[33,46]	+	+	+	+	+	+	+	+	+	+	+	+	+	+	+	+	+	+	+	+
69	Pinobanksin 3-*O*-hexanoate ^b,c^	28.21	**282**	369.1347	[32,33,46,49]	+	+	+	+	tr	tr	+	+	+	+	+	+	+	+	tr	tr	+	+	tr	+

* Component tentatively identified; ** Glycerol substitution condition predicted according to comparison with GC-MS data; ^a^ Confirmed with standard; ^b^ Confirmed with HR-MS, MS/MS (data not shown) and/or UV; ^c^ Confirmed with references; nd—Ion not detected in applied analytical conditions; + compound detected; − compound not detected; tr—compound found in traces.

**Table 6:** The content of selected major compounds determined in propolis extracts by UHPLC-DAD (data are expressed as mg/g of extracts).

No.	Compound	Rt [min]	Sample Number																			
			1	2	3	4	5	6	7	8	9	10	11	12	13	14	15	16	17	18	19	20
1	Caffeic acid	11.02	2.94	2.91	1.90	4.43	1.59	2.19	2.09	4.73	3.80	1.93	4.01	13.86	2.15	3.22	1.67	3.72	3.91	4.06	2.30	8.59
2	Vanillin	12.41	4.34	7.49	8.96	6.64	6.47	4.26	7.66	12.01	10.14	7.60	6.82	5.48	11.69	10.75	10.40	8.64	8.17	6.18	6.12	8.83
3	Benzoic acid	13.46	10.38	9.29	15.10	14.30	10.61	5.08	13.8	60.26	24.67	22.15	15.35	18.57	39.02	25.20	51.66	36.41	45.86	20.13	42.90	6.84
4	*p*-Coumaric acid	13.81	32.50	32.25	20.04	34.70	29.44	41.84	32.8	31.87	18.9	22.92	24.39	15.31	28.62	31.14	20.10	52.83	39.28	43.61	36.97	13.69
5	Ferulic acid	14.63	6.61	8.14	10.39	11.74	8.90	2.92	5.87	37.11	17.65	15.21	13.42	6.38	20.36	26.04	17.52	27.46	21.12	15.06	18.73	5.60
6	Isoferulic acid	14.74	2.28	2.32	1.60	4.00	1.00	1.15	1.12	2.58	2.95	1.11	4.50	11.55	1.10	2.14	0.99	1.57	1.07	1.10	1.05	5.40
7	Dimethylcaffeic acid ^a^	16.40	0.15	0.75	0.49	2.24	0.20	0.24	0.22	0.47	0.17	0.19	0.11	9.60	0.17	0.13	0.13	0.13	0.27	0.16	0.16	0.43
8	Cinnamic acid	16.75	4.34	4.52	4.42	5.88	4.21	4.65	4.44	5.71	4.95	4.30	4.79	5.40	5.10	4.76	5.67	5.20	5.31	4.83	4.95	4.54
9	Pinobanksin 5-methylether ^c^	17.32	3.55	3.80	1.09	6.01	0.05	0.35	0.25	4.23	6.33	0.72	8.06	19.33	0.37	2.96	0.11	0.22	0.53	0.54	0.14	12.66
10	Quercetin	17.89	1.12	1.56	0.73	1.13	0.78	0.86	0.80	1.89	1.37	0.86	1.21	3.52	0.66	1,00	0.67	0.69	0.81	0.92	0.64	2.46
11	Pinobanksin	18.44	7.49	7.33	3.64	8.81	3.36	5.77	5.82	2.50	8.98	2.52	8.76	23.19	2.09	2.93	tr	1.97	3.49	5.47	1.43	24.63
12	Naringenin	18.92	1.15	1.26	0.51	0.85	1.54	1.21	1.30	1.43	0.73	1.34	0.80	1.55	0.61	0.72	1.54	1.00	1.36	1.85	0.72	0.79
13	Apigenin	19.26	3.24	3.74	1.65	3.55	2.87	2.20	2.04	3.70	3.97	3.57	4.52	6.36	1.63	2.83	3.34	1.57	2.90	2.84	2.06	4.90
14	Kaempferol	19.44	4.40	5.30	2.03	4.70	4.65	4.74	4.53	1.80	2.84	5.02	4.28	9.80	1.85	1.98	3.83	2.05	3.78	6.24	1.54	5.65
15	Isorhamnetin	19.72	0.81	1.40	0.39	0.48	1.50	0.19	0.15	1.09	0.94	2.98	1.61	3.21	0.37	0.87	2.73	0.31	0.67	0.60	0.70	2.20
16	Rhamnetin	20.91	0.67	0.67	0.34	0.70	0.33	0.34	0.36	0.98	0.95	0.50	0.88	2.88	0.29	0.64	0.27	0.18	0.34	0.43	0.20	1.57
17	Caffeic acid prenyl or isoprenyl ester II ^a^	21.23	2.35	2.42	1.43	2.52	0.69	0.51	0.52	6.63	3.28	1.71	5.28	10.51	1.83	3.67	1.25	2.74	3.43	1.32	2.31	18.40
18	Caffeic acid prenyl or isoprenyl ester III ^a^	21.33	0.87	0.67	0.47	1.12	0.49	1.06	0.74	0.13	0.32	0.54	0.65	0.99	0.41	0.47	0.18	0.58	0.81	1.13	0.62	0.43
19	Caffeic acid benzyl ester ^a^	21.64	1.69	2.27	1.35	5.45	0.50	0.60	0.58	4.02	4.29	0.95	4.61	19.62	0.86	1.73	0.27	1.06	1.26	1.45	0.76	11.53
20	Chrysin	21.93	11.08	11.13	4.64	16.30	2.73	6.90	6.26	14.23	20.34	4.38	15.66	43.00	3.34	7.45	0.58	1.78	3.82	6.69	1.74	35.76
21	Pinocembrin	22.12	58.34	53.84	24.65	72.76	29.38	53,00	52.69	47.57	60.38	28.22	48.17	167.71	15.95	23.92	2.35	11.94	24.49	49.26	14.44	164.32
22	Sakuranetin	22.38	20.85	20.54	9.47	17.46	22.69	19.85	18.65	7.00	14.19	29.03	17.54	tr	8.17	7.86	33.03	9.61	17.76	25.68	11.33	tr
23	Galangin	22.43	11.24	9.25	4.03	10.35	7.91	8.08	7.44	3.27	9.23	8.61	11.57	27.29	2.65	3.72	7.25	2.47	5.84	9.35	3.90	38.39
24	Pinobanksin-3-*O*-acetate ^c^	22.80	23.56	25.31	18.77	33.94	17.77	18.54	17.64	54.57	35.81	22.08	41.55	41.77	23.78	38.96	10.80	37.08	35.17	24.17	29.94	78.99
25	*p*-Coumaric acid benzyl ester ^b^	23.88	4.40	4.26	1.94	4.08	3.64	6.84	5.63	0.58	2.03	2.39	0.68	2.82	1.85	0.57	0.43	2.41	3.29	6.12	1.90	2.69
26	*p*-Coumaric acid phenethyl ester ^b^	25.06	1.99	1.72	0.10	1.18	1.69	3.44	2.16	0.10	0.79	1.32	0.06	0.75	0.09	0.08	0.02	1.25	1.75	3.30	0.78	0.73
27	*p*-Coumaric acid pentyl or isopentyl ester II ^b^	25.26	2.20	2.08	0.99	1.57	1.96	3.80	2.62	tr	0.65	1.29	0.02	0.65	0.90	0.03	tr	1.26	1.81	3.37	0.91	0.80
28	*p*-Coumaric acid cinnamyl ester ^b^	26.83	10.42	10.17	4.63	8.50	7.54	16.60	12.69	1.48	4.25	6.05	0.65	5.86	6.02	1.66	0.68	5.46	8.59	9.52	4.79	3.82
29	Pinostrobin	27.20	9.17	7.74	6.33	6.75	7.73	13.12	10.62	0.14	5.51	9.00	5.86	5.62	4.73	1.91	2.04	3.83	5.49	8.59	4.21	13.60
		**Sum**	**244.5**	**244.5**	**152.3**	**292.5**	**182.6**	**230.9**	**221.9**	**312.2**	**270.6**	**208.7**	**255.9**	**482.8**	**186.8**	**209.5**	**179.7**	**225.7**	**252.7**	**264.5**	**198.5**	**478.5**

tr—Found in traces; ^a^ Calculated as caffeic acid equivalent; ^b^ Calculated as *p*-coumaric acid equivalent; ^c^ Calculated as pinobanksin equivalent.

**Table 7:** MSSA and MRSA strains used in this work.

No.	Number	Material	Ward	Antibiogram ^1^
1	4471313	Nasal swab	Intensive care	Pen. – R, Met.– S, Clin.– S, Ery. - S
2	4475564	Nasal swab	Internal	Pen.– R, Met. – S, Clin. – R, Ery. - R
3	4476206	Sputum	Internal	Pen. – R, Met. – S, Clin. – R, Ery. – R
4	4475131	Pus	Internal	Pen. – R, Met. – S, Clin.– R, Ery. – R
5	4466686	Sputum	Surgical	Pen. – R, Met. – S, Clin. – R, Ery. – R
6	4467080	Nasal swab	Internal	Pen. – R, Met. – S, Clin. – S, Ery.– S
7	4466380	Wound	Surgical	Pen.– R, Met. – S, Clin. – S, Ery.– S
8	4466896	Nasal swab	Internal	Pen. – R, Met. – S, Clin. – S, Ery.– S
9	4468792	Pharyngeal swab	Pediatrics	Pen. – R, Met. – S, Clin. – S, Ery. – S
10	4468706	-	-	Pen. – R, Met. – S, Clin. – R, Ery. – R
11	4467076	A swab from the ear	Laryngology	Pen. – R, Met.– S, Clin.– S, Ery.– S
12	4468505	Nasal swab	Internal	Pen. – R, Met. – S, Clin. – R, Ery.– R
13	4467526	Pharyngeal swab	Cardiology	Pen. – R, Met. – S, Clin. – S, Ery.– S
14	4467804	A swab from the skin	Dermatology	Pen. – R, Met. – S, Clin. – S, Ery. – S
15	4436126	A swab from the ear	Laryngology	Pen. – R, Met. – S, Clin. – S, Ery. – S
16	4467983	A swab from the ear	Laryngology	Pen. – R, Met. – S, Clin. – S, Ery. – S
17	45300223	Blood	Pediatrics	Pen – R, Met – R, Clin – R, Ery – R
18	9935169	Wound	Dispensary	Pen.– R, Met. – R, Clin.– R, Ery.– R
19	9944662	Nasal swab	Dermatology	Pen. – R, Met. – R, Clin.– R, Ery. – R
20	8007171	Wound	Laryngology	Pen.– R, Met.– R, Clin. – R, Ery. – R
21	9572250	Wound	Internal	Pen. – R, Met. – R, Clin. – R, Ery. – R

^1^ – Identification of bacterial isolates and antibiograms were performed by Laboratory of Clinical Microbiology, University Centre for Laboratory Diagnostics, Medical University of Gdańsk Clinical Centre with Vitek2 Biomerieux system; Pen—Penicillin, Met—Methicillin, Clin—Clindamycin, Ery—Erythromycin, R—resistance, S—sensitive.
